# A digital intervention to reduce home-office workers’ sedentary behaviour: protocol for the evaluation of the Click2Move programme, a cluster randomised controlled trial

**DOI:** 10.1186/s12889-025-21598-7

**Published:** 2025-01-30

**Authors:** Judit Bort-Roig, Iris Parés-Salomón, Bette Loef, Cristina Vaqué-Crusellas, Alan Coffey, Arnau Gustems-Morral, Anna M. Señé-Mir, Izabela Luznik, Maja Pajek, Kieran P. Dowd, Anna Puig-Ribera, Karin I. Proper

**Affiliations:** 1 Faculty of Health Sciences and Welfare, University of Vic - Central University of Catalonia (UVic-UCC), Vic, Barcelona, Spain; 2 Sport and Physical Activity Research Group, Institute for Research and Innovation in Life and Health Sciences in Central Catalonia (IRIS-CC), University of Vic– Central University of Catalonia (UVic-UCC), Vic, Barcelona, Spain; 3 Centre for Prevention, Lifestyle and Health, National Institute for Public Health and The Environment, Bilthoven, The Netherlands; 4 Research Group on Methodology, Methods, Models and Outcomes of Health and Social Sciences (M3O), Institute for Research and Innovation in Life Sciences and Health in Central Catalonia (IRIS-CC), Vic, Barcelona, Spain; 5 SHE Research Group, Department of Sport and Health Sciences, Technological University of The Shannon, Dublin Road, Athlone, Co., Westmeath, Ireland; 6 Sport and Physical Activity Research Group, Sport and Physical Activity Studies Centre, University of Vic-Central University of Catalonia, Vic, Barcelona, Spain; 7 Faculty of Sport, University of Ljubljana, Ljubljana, Slovenia; 8 Department of Public and Occupational Health, Amsterdam UMC, Vrije Universiteit Amsterdam, Amsterdam Public Health Research Institute, Amsterdam, The Netherlands; 9 University of Vic– Central University of Catalonia, C. Sagrada Família, 7, Vic, Barcelona, 08500 Spain

**Keywords:** Home-office work, Hybrid office work, Digital intervention, Sedentary behaviour, Protocol

## Abstract

**Background:**

A new paradigm of hybrid working exists, with most office workers sharing their work between the office and home office environment. Working from home increases time spent or prolonged sitting, which is associated with an increased risk of chronic disease. Interventions to reduce sitting time, specifically designed for both the office and home-office environments, are required to address this growing public health issue. This study presents a protocol to evaluate the effectiveness of a digital intervention (Click2Move) to reduce sitting time and improve employees’ health and occupational wellbeing among hybrid office workers.

**Methods:**

A two-arm cluster randomised controlled trial will be undertaken among hybrid office employees. In total, 200 employees will be recruited across four companies across Europe (The Netherlands, Spain, Ireland, and Slovenia). Participants within each company will be randomly allocated to the intervention or control group at the unit/cluster level. The intervention group will receive the novel multicomponent Click2Move intervention (including environmental, organisational, and individual strategies) for 12 months, and the control groups will maintain their usual work practices. The primary outcome will be occupational sedentary time measured via activPAL^3TM^ at baseline and at 3, 6 and 12 months follow-up. Secondary outcomes will include device-based (activPAL^3TM^) and self-reported (Global Physical Activity Questionnaire and Workforce Sitting Questionnaire) physical activity and sedentary behaviour; self-reported musculoskeletal disorders (Standardised Nordic Questionnaire) and pain (Numeric Rating Scale); self-reported presenteeism and absenteeism (Health and Work Performance Questionnaire), job satisfaction (Need for Recovery scale) and fatigue (single-item 5-point Likert scale). Focus groups will be conducted with employees post-intervention. Linear mixed models, accounting for covariates, will be employed to determine the effects of the intervention. Additionally, we will perform a full process evaluation analysis.

**Discussion:**

The proposed study will offer a comprehensive evaluation of a digital intervention aimed at reducing sedentary behaviour among hybrid office workers, offering practical solutions to enhance the health, wellbeing and productivity of a growing segment of the workplace.

**Trial registration:**

ClinicalTrials.gov NCT06247228. Registered 30 January 2024.

**Supplementary Information:**

The online version contains supplementary material available at 10.1186/s12889-025-21598-7.

## Background

The importance of reducing and limiting daily sedentary behaviour (SB) has been well acknowledged in existing guidelines for promoting health in addition of achieving the physical activity (PA) recommendations for health (at least 75–150 minutes a week of vigorous PA intensity, or an equivalent combination of moderate-intensity and vigorous-intensity activity throughout the week) [1]. While being highly active (60–75 minutes/day of moderate to vigorous PA (MVPA)) may attenuate the health-related risks of high levels of sedentary time (> 8 hours/day) [2, 3, 4], they may not be fully eliminated [5]. Although PA can be undertaken in a range of domains, including occupation, transport, household or leisure settings, a very low proportion of individuals (< 5% of population) perform the minimum recommended levels of MVPA to maintain health [2]. This is particularly relevant among office workers who spend 72.5% of their working hours in sedentary time [6], with those who spend a larger proportion of their time sitting at work also spending more time sitting during leisure time [7]. Prolonged occupational SB has been associated with health and work-related outcomes, including musculoskeletal disorders [8], job satisfaction, fatigue [9], and presenteeism [10].

The COVID-19 pandemic has drastically transformed work culture towards greater flexibility concerning work location, time, and organization [11]. In Europe, approximately 40% of employees began working from home due to the pandemic [12]. Since then, the new hybrid work paradigm, combining office work and home-office work, has been predominantly used among desk-based occupations [13]. Emerging research suggests that home-office workers are more sedentary and less physically active than traditional office workers [14, 15]. This could be attributed to reductions in activity achieved through commuting to and from work, overlapping online meetings, and having fewer face-to-face social interactions [16] which result in longer sitting hours with fewer breaks [17]. Due to the increased time spent in prolonged SB when working from home, employees may be at an increased risk of developing musculoskeletal disorders [18, 19, 20], poor health and work-related outcomes such as well-being and productivity [21, 22, 23]. Therefore, occupational interventions designed to reduce and fractionate prolonged sedentary periods in home-office and hybrid workers are required [15, 24].

Recent systematic reviews have reported that multicomponent interventions, including two or more of the following aspects: (i) environmental changes (e.g., sit-stand desks or active workstations); (ii) workplace policy changes (e.g., walking strategies such as incidental movement); or (iii) information and counselling interventions (e.g., providing educational material or self-measurement activity) are most effective in reducing SB among office workers than single-component interventions [25, 26, 27, 28]. In particular, interventions that incorporate sit-stand desks appear more effective than interventions without, but they may not be suitable for hybrid jobs due to the high-cost implications [29]. Moreover, effective behavioural change strategies at the workplace are not necessarily to be feasible in this new dynamic paradigm, which implies a constant change in the social and physical environment.

Reducing and breaking up prolonged SB when working from home is an emerging yet unexplored research area. Therefore, it is important to engage stakeholders and end users in developing and evaluating tailored interventions that address the real needs of the changing work environment, through a participatory research approach. Explorative studies have identified promising strategies to support workers in the home environment through a range of behavioural strategies, such as feedback on activity patterns, self-monitoring activity behaviours, educational material, prompts, motivational messages, role modelling, awards or incentives [27, 30, 31], with the findings of these studies highlighting digital technologies as a potential mode of delivery [31]. Technological advancements not only enable the delivery of information but also mediate organizational support and social influences [32], a challenge in the home office context [24]. Additionally, they provide feedback based on self-reported data and offer decision prompts through novel interfaces and devices, making interventions more adaptive to the user’s context [32], especially within the diverse landscapes of hybrid workers. In recent years, some studies have demonstrated their effectiveness on incorporating digital elements for delivering workplace strategies in reducing workplace SB and its harmful effects [27, 33], however, less is known on how digital elements may enhance interventions for reducing and breaking SB on hybrid contexts [27].

Consequently, the primary aim of this study is to evaluate the effectiveness of a digital intervention (Click2Move (C2M)) among home-office workers in reducing occupational SB. This study will also aim to evaluate changes in other PA and SB patterns (occupational behaviours and leisure time behaviours), impacts on musculoskeletal health and work-related outcomes (i.e., absenteeism, presenteeism, occupational fatigue and job satisfaction). Additionally, this study will conduct a process evaluation of the implementation of the C2M intervention.

## Methods

The present protocol has been developed and reported based on the Standard Protocol Items: Recommendations for Interventional Trials (SPIRIT) [34].

### Trial design

A multicentric, two-arm cluster randomised controlled trial (RCT) will be undertaken. Participants will be randomised by cluster to receive the intervention (C2M) or act as a unit in the control group and maintain their usual work. This study will be conducted, analysed, and reported according to the Consolidation Standards of Reporting Trials (CONSORT) statement for cluster-RCTs [35]. The trial has been registered at ClinicalTrials.gov Protocol Registration and Results System (NCT06247228).

### Study setting

Companies with home-office policies will be selected from countries of the C2M project consortium (i.e., Spain, Ireland, The Netherlands, and Slovenia) representing different geographic regions, cultures, and legislations across Europe.

### Eligibility criteria

#### Companies

Companies having different units, with 50 or more employees, and applying home-office policies will be included in the study. The list of potential companies was provided by the European Network for Workplace Health Promotion, which is also involved in the C2M project. All those companies involved in other initiatives to reduce SB will be excluded from the study.

#### Participants

Participants must be adults aged 18–65 years old with a desk-based job, working from home at least two days per week. Furthermore, participants must be able to walk without the use of an assistive device or requiring assistance from another person. Participants who are unable to read and understand the language native to the country in which they are residing, or employees with an end date for employment prior to the study completion date, will be excluded from the study. Participants using other devices for activity tracking such as a smartwatch will be eligible for inclusion.

### Recruitment

**Fig. 1:**
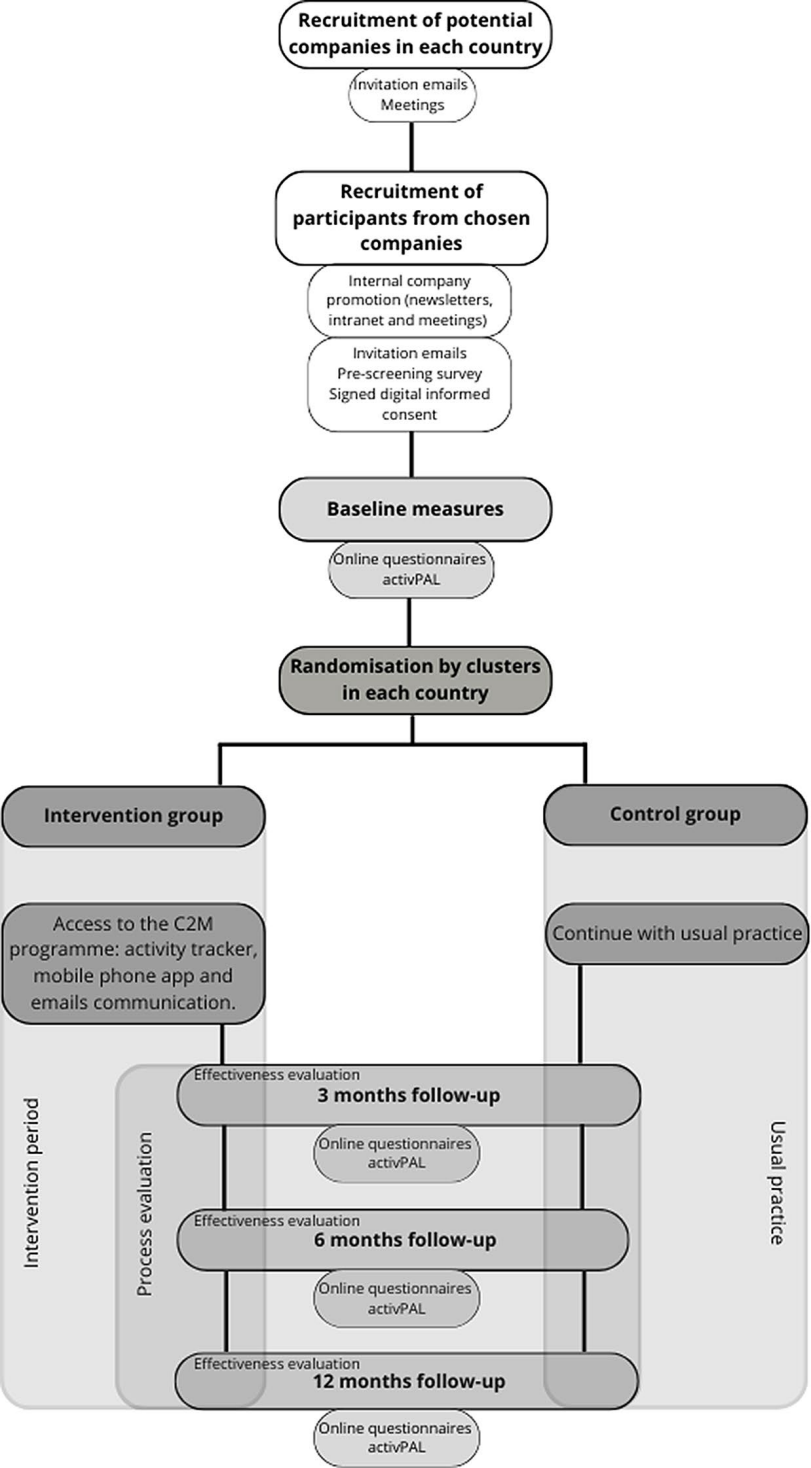
Click2Move study flow chart

Within each participating country, each national coordinator will send an initial email to potential country-representative companies from a variety of sectors in their own language with an accompanying flyer and a short company eligibility survey. The companies who express their interest to participate, and are eligible for participation, will receive an email to organise an appointment with the national research team. During this meeting, the project will be explained in greater detail and any possible doubts or issues the company may have will be discussed. If a commitment is made, the company will have access to the following promotional materials: study presentation, a flyer with the key study information and elements, study information email template and the ethical information statement. The study will be advertised by the company, through an internal newsletter and meetings, to employees that undertake hybrid working. Moreover, employees will receive an invitation email by the company manager with the pre-screening eligibility survey, the information statement, and the informed consent form. Those employees who meet the inclusion criteria and sign the informed consent digitally through the pre-screening eligibility survey, will be contacted to thank them for their interest and will be invited to a meeting to examine baseline measures. Figure 1 describes the flow chart for all elements of the C2M study.

### Allocation and blinding

After completion of the baseline measurements, participants will be randomised by clusters (units) 1:1, to either intervention or control group to reduce the risk of contamination. The randomisation will be performed by the principal investigator (JBR). Participants will be informed of the group they have been allocated to by email. For the intervention group, this email will also include the link to download the C2M App, an infographic containing a usage tutorial of the programme with frequently asked questions, and a video tutorial on the C2M programme. Only participants allocated to the intervention group will be invited for another meeting within the company, which will take place one week after receiving the group allocation email.

Given the nature of the study, it will not be possible to blind participants to their allocated group. However, the randomisation will be taken at the unit level to reduce the risk of contamination between groups (i.e. intervention and control groups will be located at different units). Measurement team members will not be blinded to group allocation.

### Sample size

The sample size was based on detecting a relevant effect on the primary outcome, sitting time during working hours. Based on an assumed mean reduction of 55.92 min/day sitting in the intervention group and a standard deviation in both groups of 90 min/day [36], a power of 80%, a two-sided alpha of 0.05, and a 20% dropout rate, a total of 200 participants across four companies at baseline will need to be recruited. Within each company, randomization in the intervention group or control group takes place at the unit level (clusters) through a hierarchic clustering between units in a company, assuming a low intra-cluster correlation coefficient (ICC) of 0.05 for the unit and 0.2 for the company. Per company, one control unit and one intervention unit will be included with the total number of participants being equal per company (*n* = 50). The total sample size will be equally distributed across both conditions (25 in the intervention group and 25 in the control group in each company in each country).

### Intervention

#### Development

The C2M intervention has been developed from a participatory research approach, with academics working alongside other stakeholders [37]. The development process of the C2M intervention includes a systematic review [27], two qualitative studies, including employers and employees [38] and a Delphi study with experts on occupational health and PA promotion [31]. Moreover, C2M followed the new Medical Research Council (MRC) framework on the development of complex interventions to improve health [39, 40]. Firstly, a systematic review and meta-analysis was conducted to examine the effectiveness of digital workplace interventions to reduce time spent in SB in office workers [27]. Nineteen randomised controlled trials were included in the systematic review, eleven of which were used in the meta-analysis. A wide range of digital elements and working strategies were described among the included studies. Delivery of information and educational materials through text messages, e-newsletters, websites or videos for increasing knowledge and awareness were the most common digital workplace strategy. Scheduled prompts to break SB or participate in PA delivered via computer screens and mobile phone applications were the second most common digital workplace strategy. The results of the study indicated that multicomponent digital interventions, including elements at organisational, environmental, and at the individual level are effective to reduce workplace SB, presenting a reduction of 29.9 min/8 h workday [27].

In addition, the perspectives of managers (*n* = 20) and employees (*n* = 51) across three countries, namely Ireland, Spain and The Netherlands were gathered through semi-structured interviews, allowing for an in-depth perspective on a multinational basis and was guided by the COM-B model [38, 41]. The interviews were conducted online, and a reflexive thematic analysis approach was adopted to analyse the data and develop themes and sub-themes. Both employees and managers highlighted the need for a top-down approach to the intervention, whereby organisational support was provided to employees, which would create the freedom and opportunities to reduce SB and engage in PA during work. Employees highlighted the need for a flexible intervention, which catered for the wide range of fitness levels and interest that exist within an organisation, with the provision of a range of time-efficient exercises that employees can chose from identified as a viable approach to reduce and break up SB. The need for a multicomponent intervention, which incorporates an educational element on the health benefits of SB, was also highlighted by both employees and managers. Finally, managers highlighted the possibility of leveraging infrastructure put in place during the Covid-19 pandemic, such as online educational provision or the use of digital platforms such as mobile phones for supportive messaging, to further support those working from home.

Lastly, a modified Delphi study to reach experts’ consensus on the most feasible strategies and the most usable digital elements as a delivery method to reduce SB in home-office context was performed [31]. Work strategies and digital elements, which were selected from a scoping review of workplace PA strategies across Europe [42], the previous systematic review [27] and the needs assessment [38], were classified according to the Behaviour Change Wheel (BCW) and the Behavioural Intervention Technology (BIT) elements. Eighteen feasible work strategies were identified, with feedback on activity progress and goal achievement, creation of an action plan or giving information to increase awareness and knowledge being the highest ranked. An additional 16 digital elements were identified as useful for inclusion within the intervention, including the use of wrist-based activity trackers, app interface in mobile phones or gamification features.

To refine and improve the C2M intervention, we conducted a single-group pilot study over a one-week period. Twenty-one participants across the four countries involved in the C2M project participated in the study. The usage, usability, acceptability, perceived usefulness, and satisfaction of the C2M intervention were assessed through four 5-point Likert scales and three open ended questions. Participants used the C2M App for an average of 4 days for 8 h each day. The App was considered as acceptable, and easy to use. Additionally, the activity tracker was perceived to improve the App information. Participants perceived the contents of the programme as “quite a bit useful”, with the exception of cooperative challenges, considered not useful/not useless. Participants were satisfied with the programme, with the awareness about their own activity patterns being the most liked item, while connection issues between the activity tracker and the application were the largest issues identified. Consequently, post-test modifications were performed to address the reported major issues.

**Fig. 2:**
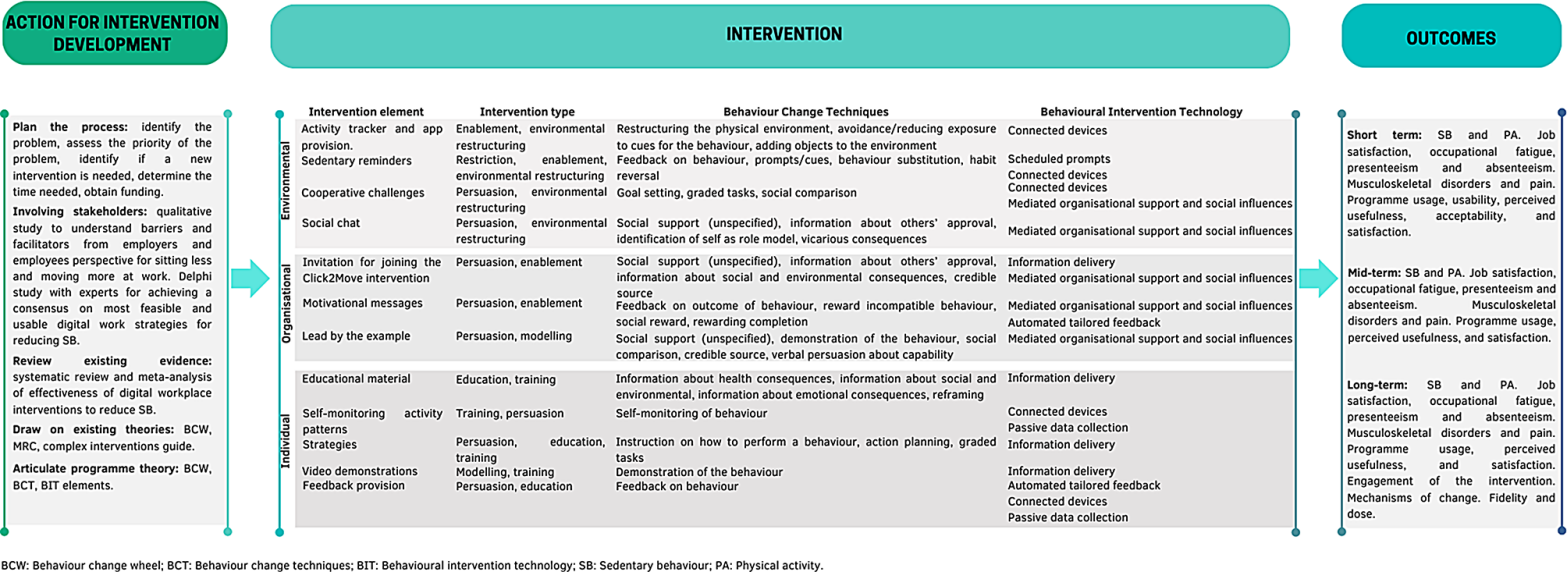
Logic model of the Click2Move intervention development

Figure 2 provides the logic model of the C2M intervention development. For each element of the intervention, the intervention type was identified according to the intervention functions from the BCW [43], the Behaviour Change Techniques (BCT) using the BCT’s Taxonomy v1 [44], and the digital elements applying the BIT classification [32].

#### Components

**Fig. 3:**
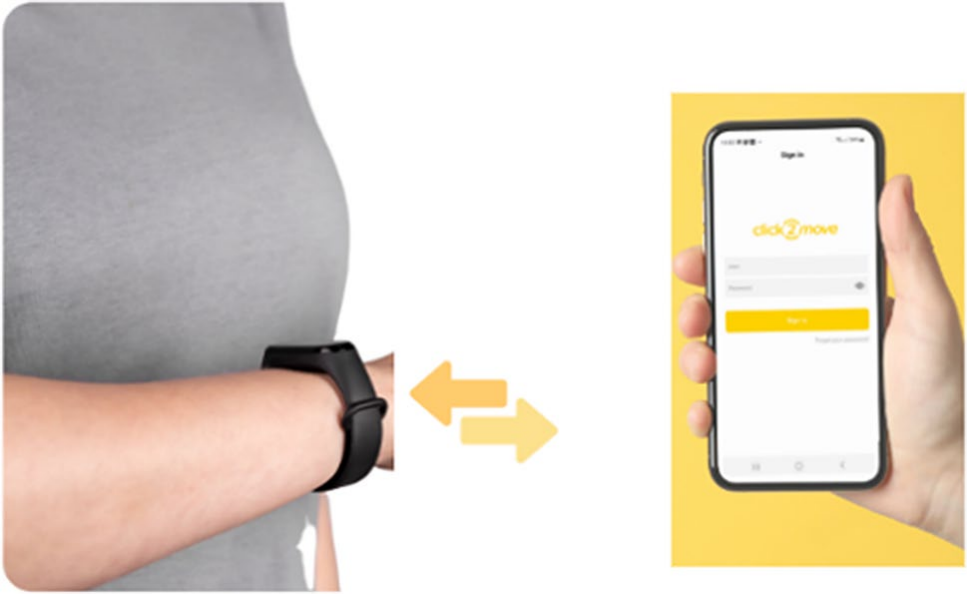
The wrist-worn activity tracker initialized with the C2M App

The C2M intervention is a multicomponent program addressing multiple levels (i.e. the environmental, organisational and individual level) aiming to reduce sitting time during working hours. The intervention includes a mobile phone application (C2M App) available in five European languages: English, Spanish, Catalan, Dutch, and Slovenian, alongside an activity tracker (Fig. 3), comprising environmental, organisational, and individual implementation levels.

The C2M App is compatible with both Android and iOS platforms and connects to an activity tracker via Bluetooth. The App receives accelerometer data and interprets it using JS libraries within React Native. Furthermore, it communicates through an API hosted on a server within the C2M host institution. The activity tracker is a waterproof wristband (1810G Monitor Smart Band, Joint Chinese Ltd., Guangdong, CHN; dimensions = 42*19*11.2 mm main body; 204.6*16.8 mm). It operates using a durable 110mAh lithium battery with a built-in USB charger and is equipped with a triaxial accelerometer sensor.

The *environmental level* is comprised of a person’s situation or environment that encourages them to break their sitting time at work [45]. Activity tracker and App provision, sedentary reminders, cooperative challenges, and social chat are the environmental elements that will be part of this level.

**Fig. 4:**
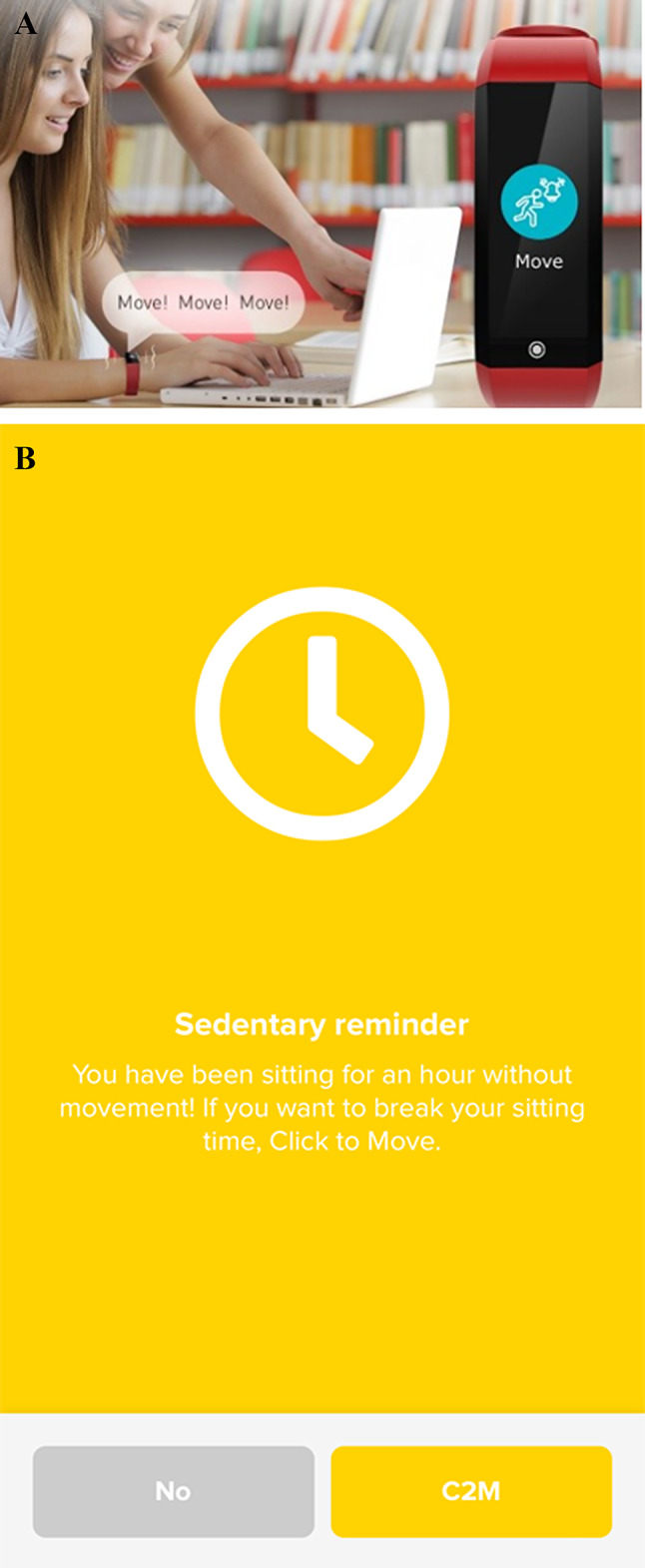
**a**. Activity tracker sedentary remainder (image provided and adapted from Joint Chinese Ltd.)**b**. C2M app sedentary reminder

The activity tracker, apart from tracking activities, provides sedentary reminders through vibrations and a message displayed on the screen of the wristband. Every uninterrupted hour without moving from the chair will result in the alert/message being displayed. After receiving the sedentary reminder on the activity tracker (Fig. 4a), the user will enter the C2M App, where a message will appear inviting them to perform a suggested active break (or strategy) (Fig. 4b). The user will be able to accept the strategy (by agreeing to complete the strategy) or to skip it.

**Fig. 5:**
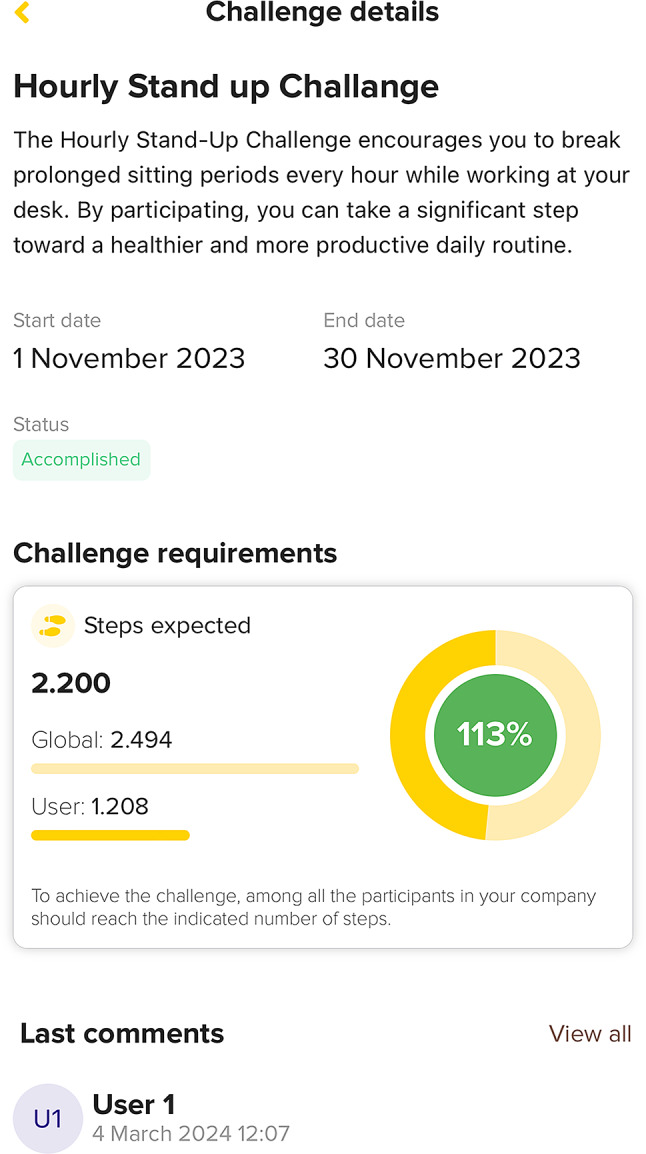
Challenges and social chat

The C2M App will offer monthly cooperative challenges for all participating company employees on the “Challenges” page. Examples include reaching a step goal or limiting the number of sedentary reminders received. Each challenge includes a title, description, and duration, as well as collective and individual achievement tracking. Each challenge will include a “social chat” where users will be able to share their experiences with other employees (Fig. 5).

The *organisational level* includes social pressure, social norms, social support, policies, rules, regulations, interaction with colleagues, and modelling that can cause individuals to change their behaviour [45, 46]. The C2M programme follows a top-down approach, involving the company in the dissemination, recruitment, and implementation phases. In addition, a monthly feedback survey and motivational messages are also strategies to be implemented at this level to provide further opportunities to break up prolonged SB.

**Fig. 6:**
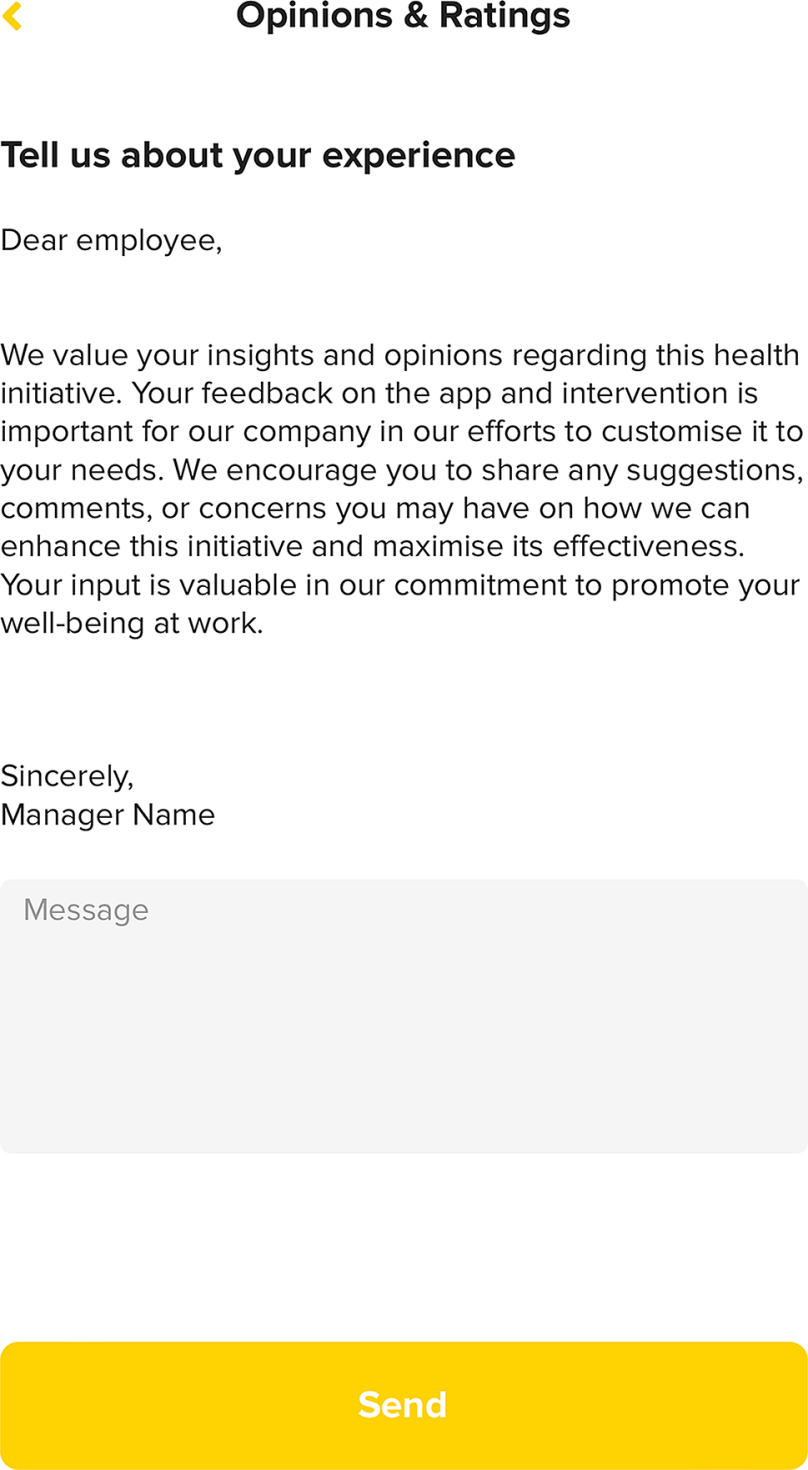
Suggestion box

**Fig. 7:**
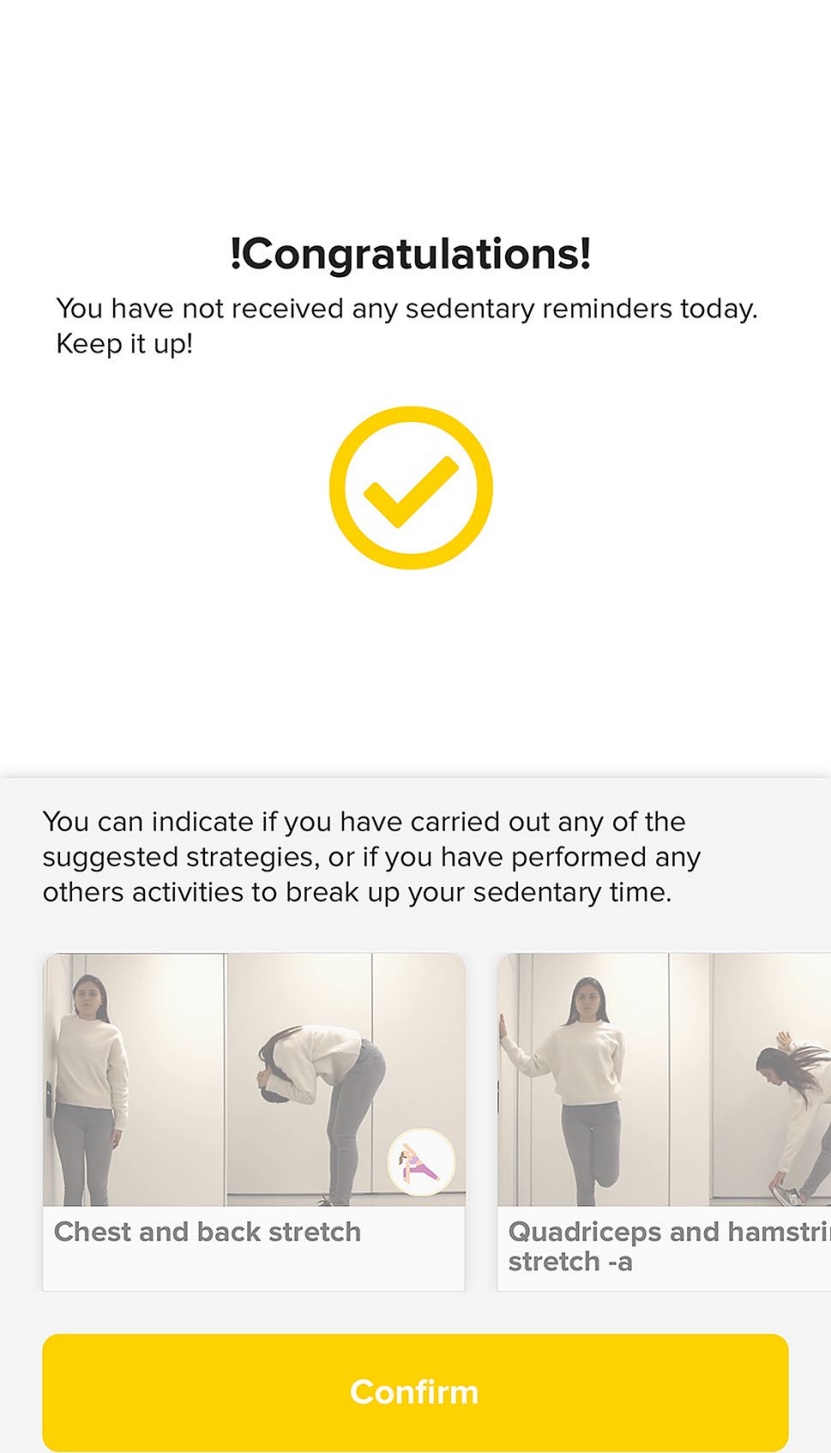
Motivational message

Specific company actions include (a) internally communicating the C2M initiative (e.g., through face-to-face or virtual presentations), (b) sending invitation emails for participation to employees, and (c) leading by example in the social chat of the App to promote engagement (i.e., managers demonstrating to employees the active breaks they are engaging in). Additionally, the C2M program will feature a permanent suggestion box on the “Profile” page of the App (Fig. 6) and monthly short surveys to gather participant feedback. Participants will also receive motivational messages related to challenge achievements and for avoiding prolonged periods of sedentary time (Fig. 7).

The *individual level* considers elements to enhance the individual’s adoption and maintenance of the behaviour (e.g., knowledge, education) [47], such as self-monitoring and feedback provision, active break strategies together with demonstrative videos and educational material.

**Fig. 8:**
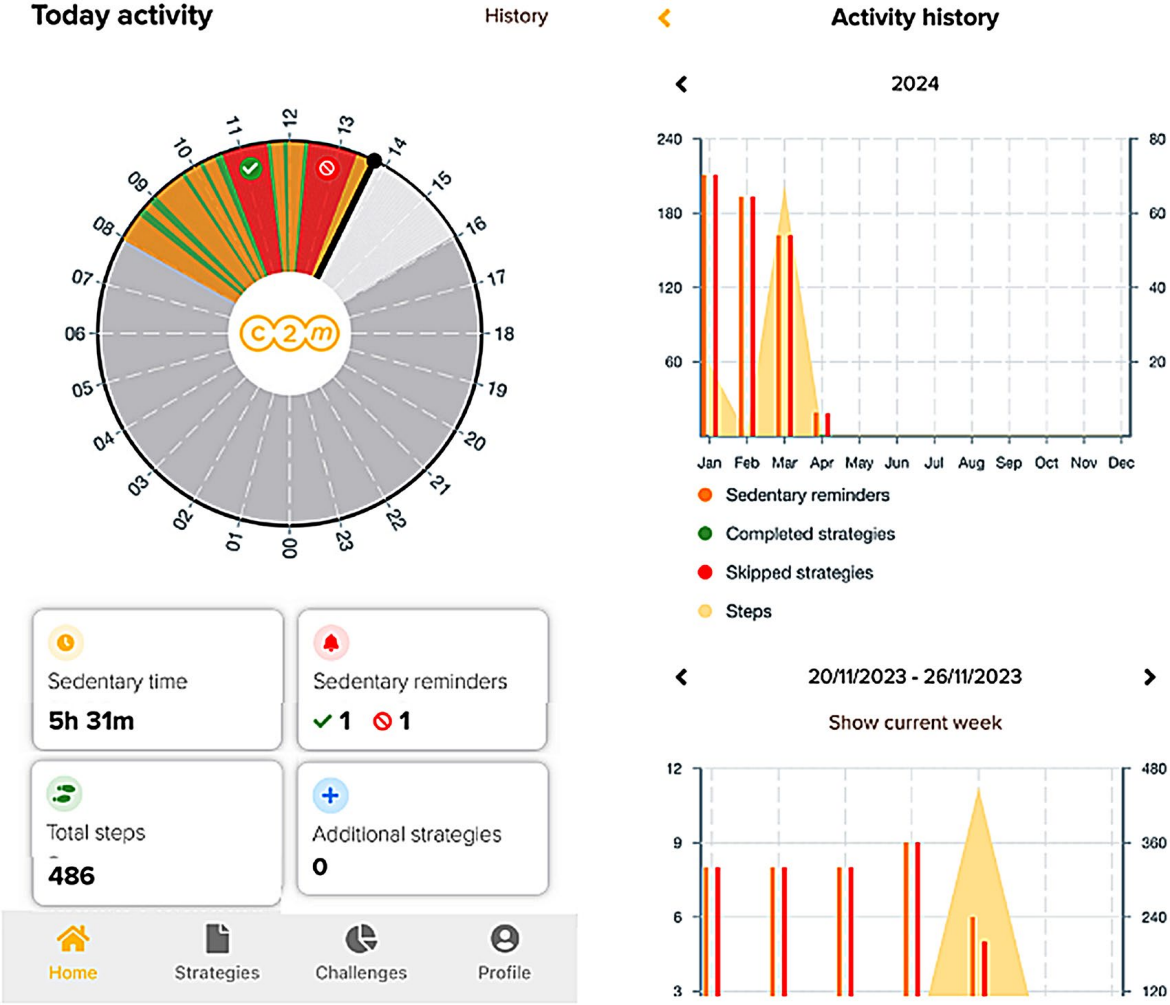
Self-monitoring feature

Users will self-monitor their working hours activity through the activity tracker and real-time feedback will be displayed on the “Home” of the C2M App. This includes metrics such as step count, time spent sedentary, status of sedentary reminders (accepted or declined), and completion status of additional strategies chosen by users without reminders. This data will also be visualized in a time continuous loop with each segment differentiated by color: green for activity time, yellow for sedentary time with bouts of less than one, and red for longer periods of sedentary time (> 1 hour). Furthermore, users can access their activity history through the “History” page (Fig. 8).

**Fig. 9:**
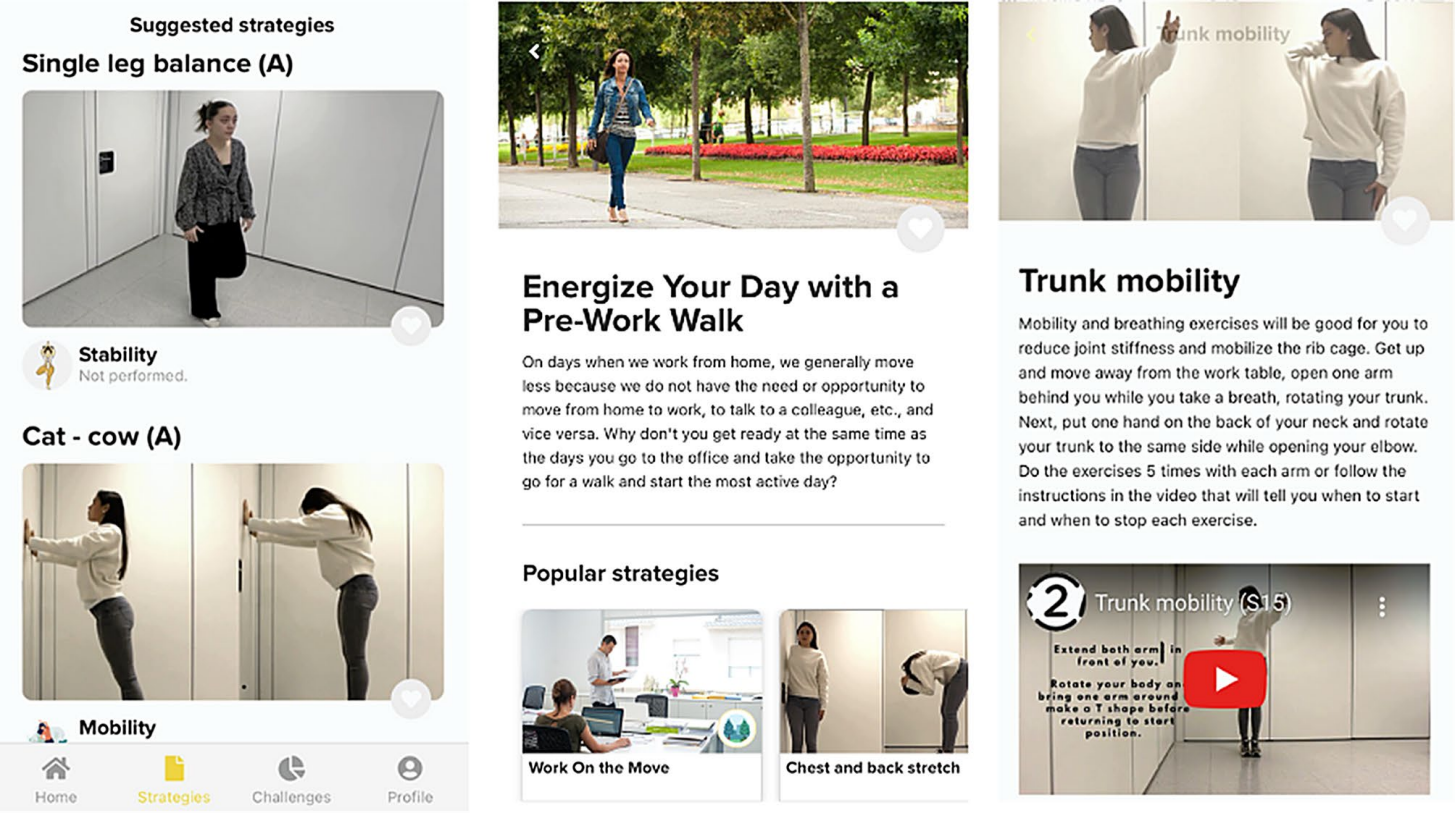
List of strategies

A list of general strategies (e.g., walk during the meetings) and active break strategies covering the following wide range of exercise types (i.e. walking, stretching, strengthening, mobility and stability) will be available on the “Strategies” page of the App (Fig. 9). Participants will be able to *like* them, and the more popular strategies will appear as preferred exercises. Each strategy will include a title, an image, a description, and a video demonstration on how to implement/complete them. Additionally, the active break strategies will be randomly suggested on the sedentary reminders message.

Finally, participants will receive monthly emails with educational material (i.e. infographics) with the aim of increasing participants’ knowledge and awareness for breaking prolonged periods of uninterrupted sedentary time. Moreover, in the description of each strategy, the benefits of implementing that strategy will be provided.

#### Intervention arm

**Fig. 10:**
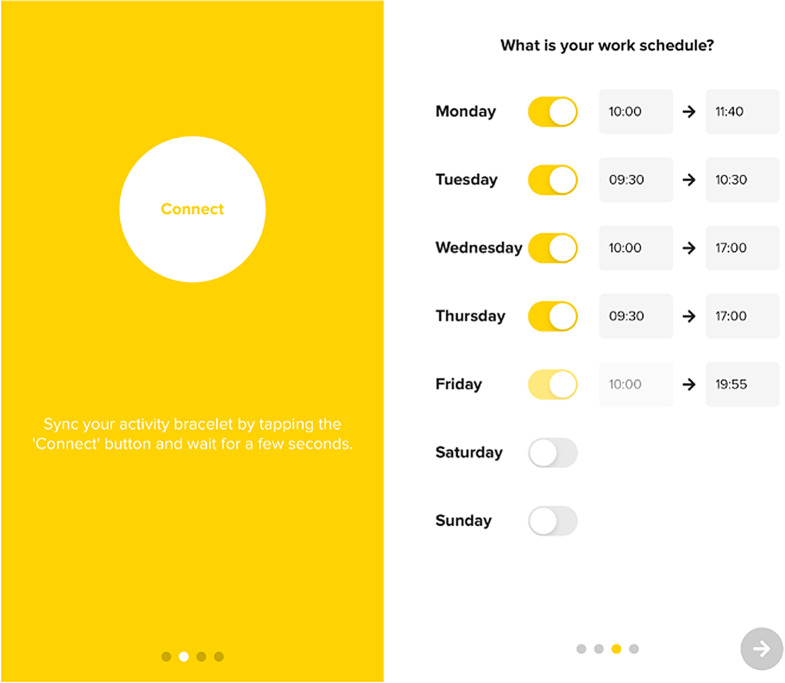
Synchronization and working hours settings

Participants from the intervention group will be invited to a face-to-face meeting to provide them with an activity tracker, and to provide a printed tutorial outlining how the App should be used. They will be asked to download the C2M App on their own mobile phone and wear the activity tracker during working hours for one year. Before the meeting, participants will be registered from the C2M App back office by the representative researchers in each country. During the meeting, the researchers will explain and demonstrate (a) how to initialise the App through the App wizard that will guide the user through the activity tracker synchronization, setting their work hours (Fig. 10), and (b) the wide range of App functionalities.

#### Control arm

Control group participants will not receive any device, nor will they have to download the mobile phone App. They will be asked to maintain they regular behaviours at work during the study period.

### Data collection

Data will be collected at four time points for both the intervention and control group participants: at baseline (T0), short term (after 3 months, T1), medium-term (6 months, T2), and long-term (12 months, T3). Measurements consist of online self-report questionnaires, an activPAL^3TM^ monitor (PAL Technologies Ltd., Glasgow, UK) and focus groups. See schedule of the enrolment, intervention, and assessments in Table 1.

The online questionnaires will be delivered through the REDCap (Research Electronic Data Capture) platform via email prior to the first appointment. The researcher will ensure that participants have completed the questionnaires online; otherwise, they will be asked to complete the questionnaires during the appointment. One week after sending the email, trained researchers will provide participants with the activPAL^3TM^ device and the diary log, along with an activPAL^3TM^ video and a hard copy tutorial.

#### Socio-demographic and home-environment measures

At baseline, participants will be asked for their age, gender, anthropometrical measures, educational level, general working conditions, home-working environment, mode of commute to and from work (on office working days), and behavioural habits (i.e. sleep conditions, and tabaco and alcohol consumption).

#### PA and SB outcomes

The activPAL^3TM^ will be used to measure and quantify the occupational and leisure PA and SB of employees, including sitting, standing, and stepping time, number and duration of sedentary bouts, sedentary to non-sedentary transitions, light Intensity PA and MVPA. The activPAL device is a valid measure to quantify body posture and activity patterns during free-living conditions in a range of populations, including healthy adults [48]. The device will be attached to the midline of the anterior aspect of the participants’ right thigh using a flexible nitrile sleeve and a transparent film (10 × 10 cm of hypoallergenic Tegaderm™ Foam Adhesive Dressing). Participants will wear the activPAL^3TM^ for 7 consecutive days, 24 h/day. Additional dressings and instructions on how to reattach the device will also be provided. Furthermore, participants will be asked to record their daily wake time, bedtime, working hours, working location (office vs. home-office), the mode of commute, PA context and any monitor removal time through the activPAL^3TM^ diary. Data will be processed using activPAL^3TM^ Professional Software™ (version 7.2.32), Microsoft Excel 2010 (Redmond, WA, USA), and MATLAB v8.4 (MathWorks^®^, Natick, MA, USA), following previously published procedure for data reduction and cleaning [49, 50, 51].

PA will also be self-reported using the Global Physical Activity Questionnaire (GPAQ), a valid and reproducible questionnaire [52]. The GPAQ questionnaire contains fifteen questions regarding PA in three domains (occupational PA, PA for transportation to and from places and PA during recreational activities). Self-reported SB will be measured through the Workforce Sitting Questionnaire (WSQ), which has previously been demonstrated to have excellent test-retest reliability and acceptable validity [53]. Five items will assess sitting time on working and non-working days in a range of domains (a) traveling, (b) at work, (c) watching television, (d) using a computer at home (outside of work), and (e) other leisure activities (but not including watching television or using computer).

#### Health-related outcomes

Health-related outcome will be operationalised by musculoskeletal symptomatology and pain intensity. Musculoskeletal symptomatology will be measured through the Standardised Nordic Questionnaire (SNQ), while pain intensity will be measured using the 11-point Numeric Rating Scale (NRS). The SNQ is an internationally self-administered questionnaire, designed for the exploration and identification of musculoskeletal symptoms, primarily in the working population. The general questionnaire consists of 8 questions with dichotomous and Likert scale (4 to 5 levels of response) responses. In all questions, it is necessary to specify for each of the 8 body parts included: neck, shoulders (differentiating between the right and left), back (differentiating between the upper and lower part), elbow-forearm (differentiating between the right and left), hand-wrist (differentiating between the right and left), hip/thigh, knees, and ankles/feet. The questions refer to the presence of discomfort, both in the last 12 months and in the last 7 days; the duration of discomfort and each episode of pain in the last 12 months; the duration of temporary disability derived from discomfort in the last 12 months; the need for treatment for the discomfort presented in the last 12 months; the intensity of discomfort present in the last 7 days; and finally, the cause of discomfort in the last 7 days. The SNQ has been demonstrated as a valid, reliable and feasible tool that enables the identification of musculoskeletal problems among different anatomical areas in epidemiological and ergonomic studies [54]. The NRS consist of a numeric version of the visual analogue scale, with a horizontal line labelled from zero to ten, with zero being an example of someone with no pain and ten being the worst pain possible. The NRS has shown high sensitivity, reliability, and validity to identify pain intensity [55].

#### Work-related outcomes

Presenteeism and absenteeism will be assessed with seven questions from the Health and Work Performance Questionnaire (HPQ) [56]. The HPQ demonstrated good validity [57], while it has also demonstrated strong reliability and sensitivity to change [56]. Four open questions on the number of days and hours participants work the past 7 days, the number of hours the employer expects employees to work in the past 7 days or in a typical 7-day week, and the number of hours participants work in the past 4 weeks will be used to score the absolute presenteeism. The number of entire or partial days missed in the past 4 weeks for mental or physical health or for other reasons (e.g., vacations) will be used to score the relative absenteeism. A high score indicates a higher amount of absenteeism. These questions are followed by two questions that ask employers to rate working in their job on a 0–10 scale of work performance (worst to best) and rank themselves over the past 4 weeks. Absolute presenteeism will be calculated as the difference between the score for one participant the past 4 weeks and the score for the average worker in the same job. In this case, a higher score indicates lower amount of lost performance. A relative presenteeism score can be computed as the ratio of individual versus other scores.

The Need for Recovery scale has demonstrated good and consistent validity to measure occupational fatigue [58]. Additionally, the Need for Recovery scale has shown favourable test-retest reliability and sensitivity to detect change [59]. The Need for Recovery comprises of eleven items with yes/no responses. A score is calculated by adding all the scores of the individual items and transforming these into a scale from 0 to 100. Higher scores indicate a higher need for recovery after work.

Job satisfaction will be measured using a single-item 5-point Likert scale [60], where 1 is extremely dissatisfied and 5 extremely satisfied. The standard satisfaction scale ranging from 1 to 5 will be converted into 0 to 100 scales using the formula: AdjSS = 100 x [stsSS-1] / [5 − 1], where adjSS and stdSS will be “adjusted satisfaction score” and “standard satisfaction score”, respectively. With the new scoring method, job satisfaction falls into 5 categories: “extremely dissatisfied” ([adjSS: 10–29], “dissatisfied” [30–49], “generally satisfied or not” [50–59], “satisfied” [70–89], and “extremely satisfied” [90–100] [61].

#### Process evaluation

The process evaluation will be performed to understand the intervention groups’ experiences, as well as to check if the intervention has been implemented as expected. The process evaluation will include a range of evaluation approaches, including checklists, engagement data, questionnaires, and focus groups. The process evaluation of the C2M project will follow the guidance on “Process evaluation of complex interventions” from the MRC guidance [62]. This framework emphasises the dynamic nature of relationships between implementation, mechanisms of impact and contextual factors.

The implementation will lead to capture the programme fidelity (whether the intervention is delivered as intended), dose (the quantity of intervention implemented), and reach (whether the intended audience meets the intervention, and how). The national coordinators will assess the fidelity and dose of the intervention through two checklists (Additional File 1). The fidelity checklist will contain all the intervention elements with a brief explanation of each one, and team members will have to complete whether each of the elements has been implemented as expected and any changes that have been made. The dose checklist will include the list of all strategies, challenges and educational material delivered, the data and duration of which it is intended to be delivered. The reach will be assessed through the back office of the C2M App, in which we will be able to see the number of steps, the sedentary reminders received, the sedentary time, the completed and skipped strategies, the additional strategies performed, the ongoing challenges, and the accomplish or failed challenges.

The mechanisms through which the C2M programme bring about change will be assessed to understand how the effects of the specific intervention elements occur and how these effects might be replicated by similar future interventions. To evaluate the programme mechanisms of impact and the contextual factors, the intervention group participants will be asked to complete self-reported questionnaires and to participate in a focus group on their experiences with the different intervention components and the behavioural change adoption and adherence for impeding or enhancing employees’ behavioural change.

The self-reported questionnaire will be delivered through the REDCap platform at 3 months, assessing the programme usage, usability, acceptability, perceived usefulness, and satisfaction through 5-point Likert scales (Additional File 2). In addition, at 6- and 12-months programme usage, perceived usefulness and satisfaction of the intervention components will be assessed. The usage of the activity tracker and C2M App will be measured through three questions with responses scoring from ‘always’ to ‘never’. Usability will be measured by the 10-item System Usability Scale [63], from ‘strongly agree’ to ‘strongly disagree’. The acceptability questions includes if the information is a) interesting, b) credible, c) easy to understand, d) new acknowledgment, e) relevant, f) excessive information provided, g) met expectations, h) social support consequences, i) behavioural change consequences, and j) self-awareness. The items will be assessed with the 5-point Likert scale ranging from ‘strongly agree’ to ‘strongly disagree’. Activity tracker acceptability, in addition to the C2M App information, will include questions related to the value, credibility personal relevance of the App information, awareness of own PA behaviour and ease of use of the tracker data and the App. The perceived usefulness will be measured by asking participants how useful they found each specific element/strategy with response option from ‘very useful’ to ‘not at all useful’. Finally, participants’ satisfaction will be measured through two 5-point Likert scale questions, one for the C2M App and the other for the activity tracker, ranging from ‘very satisfied’ to ‘very unsatisfied’.

Focus groups will be conducted at the end of the intervention. The mechanisms of impact of the intervention will be assessed through questions about the perceived effects of the intervention on SB, PA, health, work or social relationships, the components that worked and did not work and factors that have made it work or not, if intervention had any unintended consequences and if the participants would recommend the intervention. The contextual factors will be examined through questions about whether it was easier to implement the intervention at home or in the office, if the same components for reducing SB worked differently in the two contexts, how contextual factors (i.e., setting, colleagues, housemates or other aspects) effect implementation and outcomes, and to what extent participants changed their occupational behaviour while working from home and in the office. Sessions at each cluster will be facilitated by the national team using a semi-structured guide, with 6–10 participants per group.

**Table 1:**
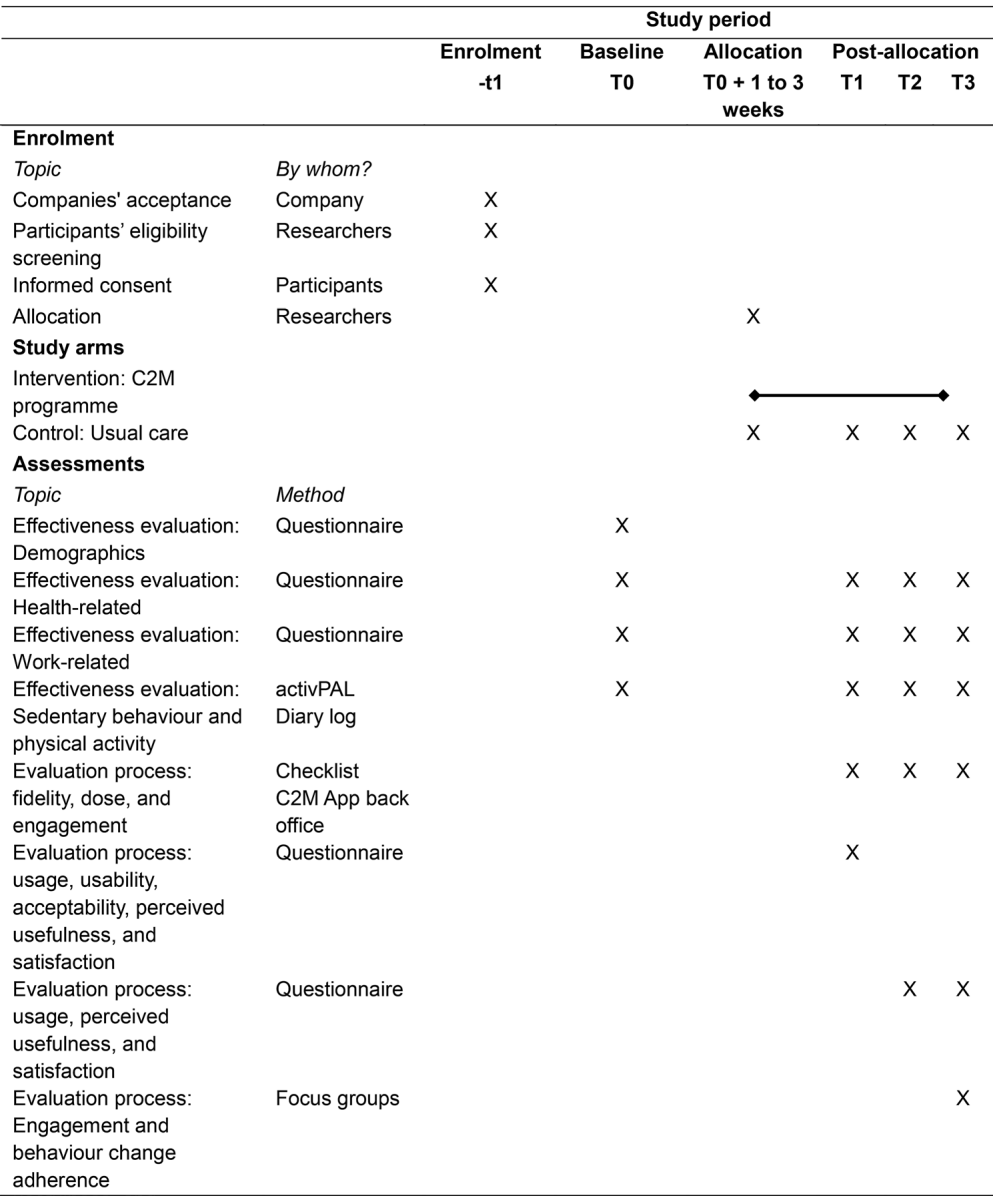
Schedule of the enrolment, intervention and assessments

### Data management

Pseudonymised data will be collected by each country and appropriately stored centrally at the REDCap platform; a secured server located at the UVic-UCC in Spain. The data management plan aligns with the FAIR data principle for its data management approach (Findability, Accessibility, Interoperability, and Reusability). Data management also aligns with all EU General Data Protection Regulations (GDPR).

### Data analysis

Data will be assessed for normality using the Kolmogorov-Smirnov method, with manual inspection of histograms and box plots for the whole group and within clusters. Descriptive characteristics on the main variables measured at baseline will be described using frequencies, mean and standard deviations and median and interquartile range as appropriate. Independent samples t-tests (or the non-parametric Mann-Whitney U test) will be used to determine differences between clusters at baseline. Linear mixed models, accounting for any covariates (i.e., country, gender, age etc.), will be employed to determine the effects of the intervention. Effect sizes will be calculated by dividing the difference in group means by the average standard deviation of the pooled data [64]. All statistical analysis will be conducted using SPSS (v 28, IBM, Chicago, IL, USA).

For the process evaluation, questionnaire data will be summarised using frequency counts and means (± standard deviation) where appropriate. Audio-recordings of focus groups will be transcribed verbatim and analysed using the principles of Thematic Analysis [65]. MAXQDA 2024 (HRB 78781, AG Berlin Charlottenburg, Germany) will be employed for the qualitative analysis.

## Discussion

Recent research on PA and occupational health has mainly focused on reducing SB among office workers due to its adverse effects on health and work-related outcomes [66, 67].This is the first article that describes the protocol for a RCT aimed at evaluating the effectiveness of a digital multicomponent intervention in reducing occupational SB among hybrid workers and its impact on employee’s musculoskeletal health, presenteeism, absenteeism, job satisfaction and occupational fatigue. Additionally, this study includes a process evaluation to understand the mechanisms of behavioural change, the programme fidelity, and participants engagement.

Previous research has highlighted the potential of digital interventions to complement occupational health interventions to reduce SB and mitigate its negative effects [27, 68]. To date, few studies have specifically targeted the home environment, which represents an emerging field of research [23, 69, 70], and none of them have integrated technological elements targeting multiple levels [27]. Given the increase in sedentary time when working from home, and the challenges in reaching employees with health initiatives in the home office environment, the development of new and effective technological interventions is essential. The C2M programme is a novel intervention using a participatory research approach, which applies potentially transferable digital work strategies to hybrid worker settings across four countries with diverse European cultural approaches, policies, and societal needs [31, 38]. Furthermore, the detailed description and classification of the multicomponent elements of the intervention, as outlined in the present protocol, will enrich the growing field of research by informing the characteristics and elements of digital interventions aimed at reducing SB in the home-office context. The BCT identification via the proposed process evaluation will facilitate comparison of interventions, as well as enhance the future replication of the most effective elements of the intervention [33]. Recent studies also examined employees perceptions and priorities when working from home [24], and existing evidence to identify effective strategies for reducing SB in an office environment, potentially offering novel insights applicable to home-office contexts [30].

In conclusion, the findings from the RCT described in this protocol will present valuable insights that can inform policy guidelines and the development of future digital interventions targeted at mitigating SB and addressing its associated health and work-related impacts within the dynamic work environment. This is especially pertinent in both office and home-office settings among hybrid workers, which signify an emerging focal point for research and policies. Additionally, knowledge gained from the process evaluation will be useful for further improvements in the implementation and thereby the effectiveness of the intervention.

## Electronic supplementary material

Below is the link to the electronic supplementary material.

Supplementary Material 1

Supplementary Material 2

Supplementary Material 3

## Data Availability

No datasets were generated or analysed during the current study.

## Abbreviations

PA: Physical activity
SB: Sedentary behaviour
SPIRIT: Standard protocol items: recommendations for interventional trials
RCT: Randomised controlled trial
CONSORT: Consolidation standards of reporting
UVic-UCC: University of Vic– Central University of Catalonia
C2M: Click2Move
ICC: Intra-cluster correlation coefficient
MRC: Medical research council
BIT: Behavioural intervention technology
BCW: Behavioural change wheel
BCT: Behavioural change techniques
REDCap: Research electronic data capture
GPAQ: Global physical activity questionnaire
WSQ: Workforce sitting questionnaire
SNQ: Standardised Nordic questionnaire
NRS: Numeric rating scale
HPQ: Health and work performance questionnaire
GDPR: General data protection regulations

## References

[1] Bull FC, Al-Ansari SS, Biddle S, Borodulin K, Buman MP, Cardon G, et al. World Health Organization 2020 guidelines on physical activity and sedentary behaviour. Br J Sports Med. 2020;54(24):1451–62. 33239350 10.1136/bjsports-2020-102955 PMC7719906

[2] Ekelund U, Steene-Johannessen J, Brown WJ, Fagerland MW, Owen N, Powell KE, et al. Does physical activity attenuate, or even eliminate, the detrimental association of sitting time with mortality? A harmonised meta-analysis of data from more than 1 million men and women. Lancet. 2016;388(10051):1302–10. 27475271 10.1016/S0140-6736(16)30370-1

[3] Stamatakis E, Gale J, Bauman A, Ekelund U, Hamer M, Ding D. Sitting time, physical activity, and risk of mortality in adults. J Am Coll Cardiol. 2019;73(16):2062–72. 31023430 10.1016/j.jacc.2019.02.031

[4] Ekelund U, Tarp J, Fagerland MW, Johannessen JS, Hansen BH, Jefferis BJ, et al. Joint associations of accelerometer-measured physical activity and sedentary time with all-cause mortality: a harmonised meta-analysis in more than 44 000 middle-aged and older individuals. Br J Sports Med. 2020;54(24):1499–506. 33239356 10.1136/bjsports-2020-103270 PMC7719907

[5] Matthews CE. Minimizing risk Associated with Sedentary Behavior: should we focus on physical activity, sitting, or both? J Am Coll Cardiol. 2019;73(16):2073–5. 31023431 10.1016/j.jacc.2019.02.030

[6] Prince SA, Elliott CG, Scott K, Visintini S, Reed JL. Device-measured physical activity, sedentary behaviour and cardiometabolic health and fitness across occupational groups: a systematic review and meta-analysis. Int J Behav Nutr Phys Act. 2019;16:1–5. 30940176 10.1186/s12966-019-0790-9 PMC6444868

[7] Clemes SA, O’Connell SE, Edwardson CL. Office workers’ objectively measured sedentary behavior and physical activity during and outside working hours. J Occup Environ Med. 2014;56(3):298–303. 24603203 10.1097/JOM.0000000000000101

[8] Dzakpasu FQS, Carver A, Brakenridge CJ, Cicuttini F, Urquhart DM, Owen N, et al. Musculoskeletal pain and sedentary behaviour in occupational and non-occupational settings: a systematic review with meta-analysis. Int J Behav Nutr Phys Act. 2021;18:1–56. 34895248 10.1186/s12966-021-01191-y PMC8666269

[9] Rosenkranz SK, Mailey EL, Umansky E, Rosenkranz RR, Ablah E. Workplace Sedentary Behavior and Productivity: a cross-sectional study. Int J Environ Res Public Health. 2020;17(18):6535. 32911740 10.3390/ijerph17186535 PMC7558581

[10] Brown HE, Ryde GC, Gilson ND, Burton NW, Brown WJ. Objectively measured sedentary behavior and physical activity in office employees: relationships with presenteeism. J Occup Environ Med. 2013;55(8):945–53. 23887700 10.1097/JOM.0b013e31829178bf

[11] Kunze F, Hampel K, Zimmermann S. Working from home in the Coronavirus crisis: Towards a transformation of work environments? 2020. https://econpapers.repec.org/paper/zbwcexpps/02.htm. Accessed 6 May 2024.

[12] Micaela (JRC-ISPRA) B. Telework in the EU before and after the COVID-19: where we were, where we head to Headlines. 2020. https://policycommons.net/artifacts/1950578/telework-in-the-eu-before-and-after-the-covid-19/2702347/. Accessed 6 May 2024.

[13] Milasi S, González-Vázquez I, Fernández-Macías E. Telework before the COVID-19 pandemic: Trends and drivers of differences across the EU. 2021. https://www.oecd.org/sti/telework-before-the-covid-19-pandemic-d5e42dd1-en.htm. Accessed 13 May 2024.14.

[14] Wilms P, Schröder J, Reer R, Scheit L. The impact of Home Office work on physical activity and sedentary behavior during the COVID-19 pandemic: a systematic review. Int J Environ Res Public Health. 2022;19(19):12344. 36231651 10.3390/ijerph191912344 PMC9566552

[15] Loef B, van Oostrom SH, van der Noordt M, Proper KI. Working from home during the COVID-19 pandemic and its longitudinal association with physical activity and sedentary behavior. Scand J Work Environ Health. 2022;48(5):380–90. 35470862 10.5271/sjweh.4027 PMC9527786

[16] Gilson N, Coenen P, Hallman D, Holtermann A, Mathiassen SE, Straker L. Postpandemic hybrid work: opportunities and challenges for physical activity and public health. Br J Sports Med. 2022;56(21):1203–4. 35710111 10.1136/bjsports-2022-105664

[17] European Foundation for the Improvement of Living and Working Conditions. The rise in telework: impact on working conditions and regulations. 2022. https://data.europa.eu/doi/10.2806/069206. Accessed 6 May 2024.

[18] Loef B, van Oostrom SH, Bosma E, Lifelines Corona Research Initiative, Proper KI. The mediating role of physical activity and sedentary behavior in the association between working from home and musculoskeletal pain during the COVID-19 pandemic. Front Public Health. 2022;10:1072030. 36530694 10.3389/fpubh.2022.1072030 PMC9757165

[19] Bosma E, Loef B, van Oostrom SH, Lifelines Corona Research Initiative, Proper KI. The longitudinal association between working from home and musculoskeletal pain during the COVID-19 pandemic. Int Arch Occup Environ Health. 2023;96(4):521–35. 36566457 10.1007/s00420-022-01946-5 PMC9790086

[20] Hong QN, Li J, Kersalé M, Dieterlen E, Mares A, Ahmadian Sangkar Z, et al. Work Disability and Musculoskeletal disorders among teleworkers: a scoping review. J Occup Rehabil. 2024 Mar;28:1–3. 10.1007/s10926-024-10184-0 38546953

[21] Krishnan C, Singh S, Baba MM. Effect of work from home and employee mental health through mediating role of workaholism and work-family balance. Int J Soc Psychiatry. 2024 Feb:00207640231196741. 10.1177/00207640231196741 37712681

[22] Galanti T, Guidetti G, Mazzei E, Zappalà S, Toscano F. Work from Home during the COVID-19 outbreak: the impact on employees’ remote work Productivity, Engagement, and stress. J Occup Environ Med. 2021;63(7):e426–32. 33883531 10.1097/JOM.0000000000002236 PMC8247534

[23] Tronco Hernández YA, Parente F, Faghy MA, Roscoe CMP, Maratos FA. Influence of the COVID-19 lockdown on the physical and Psychosocial Well-being and work Productivity of Remote Workers: cross-sectional correlational study. JMIRx Med. 2021;2(4):e30708. 34898665 10.2196/30708 PMC8641476

[24] Olsen HM, Brown WJ, Kolbe-Alexander T, Burton NW. Physical activity and sedentary behaviour in a flexible office-based workplace: employee perceptions and priorities for change. Health Promot J Austr. 2018;29(3):344–52. 29668070 10.1002/hpja.164

[25] Zhou L, Deng X, Guo K, Hou L, Hui X, Wu Y, et al. Effectiveness of Multicomponent interventions in Office-based workers to mitigate Occupational Sedentary Behavior: systematic review and Meta-analysis. JMIR Public Health Surveill. 2023;9:e44745. 37494100 10.2196/44745 PMC10413238

[26] Shrestha N, Kukkonen-Harjula KT, Verbeek JH, Ijaz S, Hermans V, Pedisic Z. Workplace interventions for reducing sitting at work. Cochrane Database Syst Reviews. 2018; 6(6). 10.1002/14651858.CD010912.pub4 PMC6513236 29926475

[27] Parés-Salomón I, Señé-Mir AM, Martín-Bozas F, Loef B, Coffey A, Dowd KP, et al. Effectiveness of workplace interventions with digital elements to reduce sedentary behaviours in office employees: a systematic review and meta-analysis. Int J Behav Nutr Phys Act. 2024;21(1):41. 38641816 10.1186/s12966-024-01595-6 PMC11031993

[28] Wang C, Lu EY, Sun W, Chang JR, Tsang HWH. Effectiveness of interventions on sedentary behaviors in office workers: a systematic review and meta-analysis. Public Health. 2024;230:45–51. 38503064 10.1016/j.puhe.2024.02.013

[29] Nguyen P, Ananthapavan J, Gao L, Dunstan DW, Moodie M. Cost-effectiveness analysis of sedentary behaviour interventions in offices to reduce sitting time in Australian desk-based workers: a modelling study. PLoS ONE. 2023;18(6):e0287710. 37384626 10.1371/journal.pone.0287710 PMC10309613

[30] Morton S, Fitzsimons C, Jepson R, Saunders DH, Sivaramakrishnan D, Niven A. What works to reduce sedentary behavior in the office, and could these intervention components transfer to the home working environment? A rapid review and transferability appraisal. Front Sports Act Living. 2022;4:954639. 35966113 10.3389/fspor.2022.954639 PMC9372484

[31] Bort-Roig J, Parés-Salomón I, Señé-Mir AM, Puig-Ribera A, Vaqué-Crusellas C, Proper KI et al. Click2Move programme: work strategies and digital solutions for its implementation. 2023. https://click2moveproject.com/phases/phase-1/. Accessed 17 May 2024.

[32] Huang Y, Benford S, Blake H. Digital Interventions To Reduce Sedentary Behaviors of Office Workers: scoping review. J Med Internet Res. 2019;21(2):e11079. 30730294 10.2196/11079 PMC6383116

[33] Buckingham SA, Williams AJ, Morrissey K, Price L, Harrison J. Mobile health interventions to promote physical activity and reduce sedentary behaviour in the workplace: a systematic review. Digit Health. 2019;5:2055207619839883. 30944728 10.1177/2055207619839883 PMC6437332

[34] Chan AW, Tetzlaff JM, Altman DG, Laupacis A, Gøtzsche PC, Krleža-Jerić K, et al. SPIRIT 2013 Statement: defining standard protocol items for clinical trials. Ann Intern Med. 2013;158(3):200–7. 23295957 10.7326/0003-4819-158-3-201302050-00583 PMC5114123

[35] Schulz KF, Altman DG, Moher D, Group CONSORT. CONSORT 2010 statement: updated guidelines for reporting parallel group randomised trials. BMJ. 2010;340:c332. 20332509 10.1136/bmj.c332 PMC2844940

[36] Edwardson CL, Yates T, Biddle SJH, Davies MJ, Dunstan DW, Esliger DW et al. Effectiveness of the stand more at (SMArT) work intervention: cluster randomised controlled trial. BMJ. 2018;363. 10.1136/bmj.k3870 PMC6174726 30305278

[37] Leask CF, Sandlund M, Skelton DA, Altenburg TM, Cardon G, Chinapaw MJM, et al. Framework, principles and recommendations for utilising participatory methodologies in the co-creation and evaluation of public health interventions. Res Involv Engagem. 2019;5(1):2. 30652027 10.1186/s40900-018-0136-9 PMC6327557

[38] Coffey A, Parés-Salomón I, Reckman P, Bort-Roig J, Proper K, Puig-Ribera AM et al. Click2Move Project. 2022. Cross-cultural needs analysis: Factors for reducing sedentary behaviours among home-office workers. https://click2moveproject.com/phases/phase-1/. Accessed 8 May 2024.

[39] O’Cathain A, Croot L, Duncan E, Rousseau N, Sworn K, Turner KM, et al. Guidance on how to develop complex interventions to improve health and healthcare. BMJ Open. 2019;9(8):e029954. 31420394 10.1136/bmjopen-2019-029954 PMC6701588

[40] Craig P, Dieppe P, Macintyre S, Michie S, Nazareth I, Petticrew M. Developing and evaluating complex interventions: the new Medical Research Council guidance. BMJ. 2008;337. 10.1136/bmj.a1655 PMC2769032 18824488

[41] Ojo SO, Bailey DP, Brierley ML, Hewson DJ, Chater AM. Breaking barriers: using the behavior change wheel to develop a tailored intervention to overcome workplace inhibitors to breaking up sitting time. BMC Public Health. 2019;19:1–7. 31420033 10.1186/s12889-019-7468-8 PMC6697980

[42] Väänänen I, Mas-Alòs S, Vandaele F, Codina-Nadal A, Matas S, Aumatell E, et al. Workplace physical activity practices in real life: a scoping review of grey literature for small- and medium-sized enterprises. Eur J Public Health. 2022;32(Suppl 1):i22–7. 36031820 10.1093/eurpub/ckac083 PMC9421405

[43] Michie S, van Stralen MM, West R. The behaviour change wheel: a new method for characterising and designing behaviour change interventions. Ann Behav Med. 2013;6(1):42. 10.1186/1748-5908-6-42 PMC3096582 21513547

[44] Michie S, Richardson M, Johnston M, Abraham C, Francis J, Hardeman W, et al. The behavior change technique taxonomy (v1) of 93 hierarchically clustered techniques: building an International Consensus for the reporting of Behavior Change interventions. Ann Behav med. 2013;46(1):81–95. 23512568 10.1007/s12160-013-9486-6

[45] Cane J, O’Connor D, Michie S. Validation of the theoretical domains framework for use in behaviour change and implementation research. Implement Sci. 2012;7:1–7. 10.1186/1748-5908-7-37 PMC3483008 22530986

[46] Rajkumar E, Rajan AM, Daniel M, Lakshmi R, John R, George AJ, et al. The psychological impact of quarantine due to COVID-19: a systematic review of risk, protective factors and interventions using socio-ecological model framework. Heliyon. 2022;8(6):e09765. 35756104 10.1016/j.heliyon.2022.e09765 PMC9212950

[47] Bowen DJ, Kreuter M, Spring B, Cofta-Woerpel L, Linnan L, Weiner D, et al. How we Design Feasibility studies. Am J Prev Med. 2009;36(5):452–7. 19362699 10.1016/j.amepre.2009.02.002 PMC2859314

[48] O’Brien MW, Wu Y, Petterson JL, Bray NW, Kimmerly DS. Validity of the ActivPAL monitor to distinguish postures: a systematic review. Gait Posture. 2022;94:107–13. 35276456 10.1016/j.gaitpost.2022.03.002

[49] Dowd KP, Harrington DM, Bourke AK, Nelson J, Donnelly AE. The measurement of sedentary patterns and behaviors using the activPAL^™^ Professional physical activity monitor. Physiol Meas. 2012;33(11):1887–99. 23111150 10.1088/0967-3334/33/11/1887

[50] Wu Y, Petterson JL, Bray NW, Kimmerly DS, O’Brien MW. Validity of the activPAL monitor to measure stepping activity and activity intensity: a systematic review. Gait Posture. 2022;97:165–73. 35964334 10.1016/j.gaitpost.2022.08.002

[51] Powell C, Carson BP, Dowd KP, Donnelly AE. Simultaneous validation of five activity monitors for use in adult populations. Scand J Med Sci Sports. 2017;27(12):1881–92. 27905148 10.1111/sms.12813

[52] Bull FC, Maslin TS, Armstrong T. Global physical activity questionnaire (GPAQ): nine country reliability and validity study. J Phys Act Health. 2009;6(6):790–804. 20101923 10.1123/jpah.6.6.790

[53] Chau JY, van der Ploeg HP, Dunn S, Kurko J, Bauman AE. A tool for measuring workers’ sitting time by domain: the workforce sitting questionnaire. Br J Sports Med. 2011;45(15):1216–22. 21947817 10.1136/bjsports-2011-090214

[54] Kuorinka I, Jonsson B, Kilbom A, Vinterberg H, Biering-Sørensen F, Andersson G, et al. Standardised nordic questionnaires for the analysis of musculoskeletal symptoms. Appl Ergon. 1987;18(3):233–7. 15676628 10.1016/0003-6870(87)90010-x

[55] Williamson A, Hoggart B. Pain: a review of three commonly used pain rating scales. J Clin Nurs. 2005;14(7):798–804. 16000093 10.1111/j.1365-2702.2005.01121.x

[56] Kessler RC, Barber C, Beck A, Berglund P, Cleary PD, McKenas D, et al. The World Health Organization Health and Work Performance Questionnaire (HPQ). J Occup Environ Med. 2003;45(2):156–74. 12625231 10.1097/01.jom.0000052967.43131.51

[57] Kessler RC, Ames M, Hymel PA, Loeppke R, McKenas DK, Richling DE, et al. Using the World Health Organization Health and Work Performance Questionnaire (HPQ) to evaluate the indirect workplace costs of illness. J Occup Environ Med. 2004;46(6):S23–37. 15194893 10.1097/01.jom.0000126683.75201.c5

[58] van Veldhoven M, Broersen S. Measurement quality and validity of the need for recovery scale. Occup Environ Med. 2003;60(1):i3–9. 12782740 10.1136/oem.60.suppl_1.i3 PMC1765728

[59] de Croon EM, Sluiter JK, Frings-Dresen MHW. Psychometric properties of the need for recovery after work scale: test-retest reliability and sensitivity to detect change. Occup Environ Med. 2006;63(3):202–6. 16497863 10.1136/oem.2004.018275 PMC2078147

[60] Scarpello V, Campbell JP. Job satisfaction: are all the parts there? Pers Psychol. 1983;36(3):577–600.

[61] Liu JA, Wang Q, Lu ZX. Job satisfaction and its modeling among township health center employees: a quantitative study in poor rural China. BMC Health Serv Res. 2010;10:115. 20459725 10.1186/1472-6963-10-115 PMC2907754

[62] Moore GF, Audrey S, Barker M, Bond L, Bonell C, Hardeman W, et al. Process evaluation of complex interventions: Medical Research Council guidance. BMJ. 2015;350:h1258. 25791983 10.1136/bmj.h1258 PMC4366184

[63] Brooke J. Bernard. SUS - a quick and dirty usability scale. CRC; 1996.

[64] Cohen J. Statistical Power Analysis for the Behavioural Sciences. 2nd Edition. Lawrence Erlbaum associates, Publishers; 1988.

[65] Braun V, Clarke V. Using thematic analysis in psychology. Qualitative Res Psychol. 2006;3(2):77–101.

[66] Bort-Roig J, Chirveches-Pérez E, Giné-Garriga M, Navarro-Blasco L, Bausà-Peris R, Iturrioz-Rosell P, et al. An mHealth workplace-based sit less, move more program: impact on employees’ sedentary and physical activity patterns at work and away from work. Int J Environ Res Public Health. 2020;17(23):1–10. 10.3390/ijerph17238844 PMC7730175 33260697

[67] De Cocker K, De Bourdeaudhuij I, Cardon G, Vandelanotte C. What are the working mechanisms of a web-based workplace sitting intervention targeting psychosocial factors and action planning? BMC Public Health. 2017;17:1–10. 28468687 10.1186/s12889-017-4325-5 PMC5415713

[68] Edwardson CL, Biddle SJH, Clemes SA, Davies MJ, Dunstan DW, Eborall H, et al. Effectiveness of an intervention for reducing sitting time and improving health in office workers: three arm cluster randomised controlled trial. BMJ. 2022;378:e069288. 35977732 10.1136/bmj-2021-069288 PMC9382450

[69] Olsen HM, Brown WJ, Kolbe-Alexander T, Burton NW. A brief Self-Directed intervention to Reduce Office employees’ sedentary behavior in a flexible workplace. J Occup Environ Med. 2018;60(10):954–9. 30001255 10.1097/JOM.0000000000001389

[70] Wilkerson AH, Elliott CR, McFadden NT, Abutalib N. Feasibility of using Mobile Standing desks to address sedentary behavior in flexible work environments: a mixed methods study. J Occup Environ Med. 2023;65(5):e273–8. 36701795 10.1097/JOM.0000000000002804 PMC10171101

