# Supplementary material for: A digital intervention to reduce home-office workers’ sedentary behaviour: protocol for the evaluation of the Click2Move programme, a cluster randomised controlled trial
Source: BMC Public Health. 2025 Jan 30;25:387. doi: 10.1186/s12889-025-21598-7 (PMC11783833; doi:10.1186/s12889-025-21598-7)
Supplement: Supplementary file 3 — Supplementary Material 3 [file 12889_2025_21598_MOESM3_ESM.docx]

**Process evaluation questionnaire**

**Usage**

In a typical working week, for how many days have you worn the C2M activity tracker?

On working days where you wore the C2M activity tracker, how often during your working day were you wearing the C2M activity tracker?

1. Always
2. Often
3. Sometimes
4. Rarely
5. Never

When wearing the C2M activity tracker on working days, how often did you use the C2M App?

1. Always
2. Often
3. Sometimes
4. Rarely
5. Never

**Usability**

The next questions are about the Click2Move activity tracker and the application (C2M App) you were provided. Please rate your agreement with the following statements:

| Strongly agree | Agree | Neither agree/disagree | Disagree | Strongly disagree |
| --- | --- | --- | --- | --- |

1. I would like to use the C2M App frequently in the future.
2. I find the C2M App too complex.
3. I thought the C2M App is easy to use.
4. I needed the support of a technical person to be able to use the C2M App.
5. I find the various features in the C2M App were well integrated.
6. I think there is too much inconsistency in the C2M App.
7. I think that most people would learn to use the C2M App very quickly.
8. I find the C2M App very cumbersome to use.
9. I feel very confident using the C2M App.
10. I need to learn a lot of things before I could use C2M comfortably.
11. I like the presentation and layout of the C2M App – colours, content and images.
12. It has been easy to connect the activity tracker with the App and sync the activity data.

**Acceptability**

The next questions are about the C2M App content and information (i.e., messages, strategies, feedback, educational information). Please rate your agreement with the following statements:

| Strongly agree | Agree | Neither agree/disagree | Disagree | Strongly disagree |
| --- | --- | --- | --- | --- |

1. The information is interesting.
2. The information is credible.
3. The information is easy to understand.
4. Through the information I have learned something new about my own activity at work.
5. The information is personally relevant.
6. Too much information is provided.
7. The information meets my expectations.
8. The information has helped me to support others to become more physically active.
9. I have used the information to become more active at work.
10. I have changed my opinion about my activity at work.

Do you think the use of an activity tracker in addition to the App information help to…

| Strongly agree | Agree | Neither agree/disagree | Disagree | Strongly disagree |
| --- | --- | --- | --- | --- |

1. Improve the value of the App information.
2. Improve the credibility of the App information.
3. Improve the personal relevance of the App information.
4. Increase your awareness of how active you are.

**Perceived usefulness**

The next questions are about how useful the C2M App was.

How useful have been the following programme components in helping you reduce your prolonged sedentary periods at work?

| Very useful | Useful | Not useful/Not useless | Useless | Very useless |
| --- | --- | --- | --- | --- |

How useful were the following program components in helping you reduce your prolonged sedentary periods at work?

1. Sedentary reminders through the activity tracker vibration.
2. Strategies proposed during the sedentary reminders through the C2M App.
3. Educational material sent via email.
4. Self-monitoring activity patterns through the activity tracker.
5. Activity feedback about the daily activity patterns through the home page of the C2M App.
6. Monthly and weekly activity patterns feedback through the historical of the C2M App.
7. List of strategies divided into different categories.
8. Information of each strategy (explanation and benefits of performing each strategy).
9. Video demonstration of each strategy except for general and walk strategies.
10. Social challenges.
11. Social chat during the social challenges.
12. Organisational support through the linking the company to the project.

**Satisfaction**

The last questions are related to the general satisfaction with the C2M App and the activity tracker:

| Very satisfied | Satisfied | Not unsatisfied/not satisfied | Unsatisfied | Very unsatisfied |
| --- | --- | --- | --- | --- |

In general, how satisfied are you with the C2M App?

In general, how satisfied are you with the C2M activity tracker?
